# The occurrence of disinfectant and antibiotic-resistant genes in *Escherichia coli* isolated from chickens in Egypt

**DOI:** 10.14202/vetworld.2019.141-145

**Published:** 2019-01-25

**Authors:** Waleed A. Ibrahim, Sherif A. Marouf, Ahmed M. Erfan, Soad A. Nasef, Jakeen K. El Jakee

**Affiliations:** 1 Reference Laboratory for Veterinary Quality Control on Poultry Production, Animal Health Research Institute, P.O. Box 264-Dokki, Giza 12618, Egypt; 2 Department of Microbiology, Faculty of Veterinary Medicine, Cairo University, Giza, Egypt

**Keywords:** antimicrobial resistance, chickens, Egypt, *Escherichia coli*, *qac* resistance genes

## Abstract

**Aim:**

This work aimed to determine the occurrence of antibiotic and disinfectant resistance genes in *Escherichia coli* isolated from chickens in Egypt.

**Materials and Methods:**

Organs (liver, lung, heart, yolk sac, and bone marrow) of 1500 chicken samples were collected from diseased chickens suffered from colibacillosis with PM findings as CRD, diarrhea and omphalitis from different governorates of Egypt as: Giza, EL-Bahira, Fayoum, El-Dakahlia, El-Ismalia, and El-Sharkia during 2015-2016. These samples were labeled and transported immediately on ice to the Reference laboratory for quality control on poultry production (RLQP). The samples were cultured onto MacConkey agar and Eosin Methylene Blue Agar. Isolation and identification of the *E. coli* were performed based on morphology, cultural, staining, and biochemical properties. Antimicrobial resistance test was carried out using disk diffusion method. The PCR employing *tetA, qacED1* and *qacA/B* were carried out for detection of these genes in isolated *E.coli*.

**Results:**

The prevalence of *E. coli* in chicken was 34%. Predominant serotypes of *E. coli* which serologically identified were O128, O111, O44, O158, and O2. Antibiotic susceptibility test of *E. coli* revealed that 100% of isolates were resistant to ampicillin, erythromycin, and sulfamethoxazole-trimethoprim, while 73.53% and 38.23% of them were sensitive for colistin sulfate and levofloxacin, respectively. Antibiotic resistance genes as *tetA* gene were tested for isolated *E. coli* and detected by incidence rate of 91.18%. *qac* resistance genes resembling as *qacED1* and *qacA/B* genes were detected in isolated *E*. *coli* 70.6% and 14.7%, respectively.

**Conclusion:**

*E*. *coli* isolated from chickens in Egypt was carried *qac* and antibiotic-resistant genes that affect the poultry industry.

## Introduction

Avian pathogenic *Escherichia coli* (APEC) unlike other normal microflora *E. coli* in poultry intestine APEC spreads into several internal organs and causes systemic fatal disease colibacillosis, which is characterized by septicemia with multiple organ lesions, typically pericarditis, airsacculitis, perihepatitis, peritonitis, and other extra-intestinal lesions [1]. In poultry farms and surrounding environment, antibiotic resistance occurs frequently and can be spread to humans through food or water chain and also by routes such as environmental contamination by poultry waste and direct interaction with animals [2]. Quaternary ammonium compounds (QACs) are cationic surface active detergents generally used for the control of microorganisms in clinical and industrial environments plus used in the disinfection of hard surfaces [3]. The last line of defense for the poultry industry could possibly be the use of disinfectants as QACs that are frequently used in environments where antibiotics are used, thus fuelling the concern of a relationship between QAC and antibiotic resistance [4]. QAC resistance genes frequently existed among *E. coli* isolates. The *qac* genes were highly associated with antimicrobial resistance phenotypes [5]. *qac* genes in Gram-negative bacteria were most frequently found in combination with genes coding for resistance to aminoglycosides, chloramphenicol, sulfonamides, trimethoprim, and β-lactams [6,7]. In the previous study, detection of the disinfectant resistant gene of aerobic bacteria in unhatched chicken eggs in Egypt was done, and the results indicate the presence of *qacED1* gene in isolated *E. coli* with incidence rate of 100% [8].

The significance of study is to explain the failure of treatment of *E. coli* infection in poultry using antibiotics and increases the infection of *E.coli* during first week of age besides that the antibiotic resistance occurs often in poultry farms and surrounding environment which can be spread to humans via food or water chain.

Hence, the present investigation aimed to study the disinfectant and antibiotic resistance genes among *E. coli* isolated from chickens in Egypt.

## Materials and Methods

### Ethical approval

Ethical approval for this study was obtained from Animal Health Research Institute of Egypt.

### Collected samples

Organs (liver, lung, heart, yolk sac, and bone marrow) of 1500 chicken samples were collected from diseased chickens suffered from colibacillosis with PM findings as CRD, diarrhea and omphalitis from different age-old and different governorates of Egypt as: Giza; 610 samples, EL-Bahira; 350 samples, Fayoum; 230 samples, El-Dakahlia; 160 samples, El-Ismalia; 120 samples, and El-Sharkia; 100 samples during 2015-2016 in winter seasons. These samples were labeled and transported immediately on ice to Reference laboratory for quality control on poultry production (RLQP). All samples were handled aseptically and examined microbiologically.

### Bacteriological examination

#### Isolation of *E. coli* by conventional method [9]

Each pooled sample was transferred to buffered peptone water and incubated for 16–18 h at 37°C. After selective enrichment, a loopful of the broth was inoculated on MacConkey agar and Eosin Methylene blue agar (Oxoid), then incubated aerobically in 37°C for 24 h. Suspected *E. coli* colonies were purified and kept for further identification.

#### Microscopic examination

Gram’s stain was prepared and used for examined suspected colonies as described by Cruickshank [10] for morphological study.

#### Biochemical confirmation

Suspected colonies were examined using different biochemical reaction including indole reaction, methyl red test, Voges–Proskauer test, citrate utilization test, catalase test, sugar fermentation test, oxidase test, triple sugar iron, and Christensen’s urea agar test according to Quinn *et al*. [9].

#### Serological identification

*E. coli* isolates were serologically identified using rapid diagnostic *E. coli* antisera Set 1 containing polyvalent and monovalent O antisera (DENKA SEIKEN Co. LTD, Japan) according to Edwards and Ewing [11].

### Antibiotic susceptibility testing

Sensitivity to 12 different groups antibacterial drugs (Ampicillin 10 µg, Amoxicillin 10 µg, Gentamicin 10 µg, Streptomycin 10 µg, Erythromycin 15 µg, Amoxi-clavulanic acid 20/10 µg, Doxycycline 30 µg, Tetracycline 30 µg, Nalidixic acid 30 µg, Levofloxacin 5 µg, Colistin sulfate 25 µg, and trimethoprim-sulfamethoxazole 1.25/23.75 µg) from Oxoid Hampshire, U K, was tested by disk diffusion method according to Quinn *et al*. [9] and Cruickshank [10]. The interpretation of the inhibition zones of tested culture was tested according to Clinical and Laboratory Standards Institute [12].

### Polymerase chain reaction (PCR) for the identification of different genes

#### Oligonucleotide primers

Primers used were supplied from Metabion (Germany) and are listed in Table-1 [13-15], and cycle condition for different primers is shown in Table-2.

**Table-1:** Primers used for sequence of partial and complete fusion gene.

Primer	Sequence	Amplified product	References
*qacED1*	TAA GCC CTA CAC AAA TTG GGA GAT AT	362 bp	[13]
	GCC TCC GCA GCG ACT TCC ACG		
*qacA/B*	GCAGAAAGTGCAGAGTTCG	361 bp	[14]
	CCAGTCCAATCATGCCTG		
*tetA(A)*	GGTTCACTCGAACGACGTCA	576 bp	[15]
	CTGTCCGACAAGTTGCATGA		

**Table-2:** Cycling conditions of the different primers.

Gene	Primary denaturation	Secondary denaturation	Annealing	Extension	Number of cycles	Final extension
*QacED1*	94°C 5 min	94°C 30 s	58°C 40 s	72°C 40 s	35	72°C 7 min
*QacA/B*			53°C 40 s			72°C 7 min
*TetA(A)*			50°C 40 s			72°C 10 min

#### DNA extraction

DNA extraction from samples was performed using the QIAamp DNA Mini kit (Qiagen, Germany, GmbH) with modifications from the manufacturer’s recommendations.

## Results

### Occurrence of *E. coli* in chickens

*E. coli* isolates showed bright pink colonies, lactose fermentable on MacConkey agar plates and showed a distinctive metallic green sheen on EMB agar plates. Biochemically, all *E. coli* suspected isolates were lactose fermenting colonies, positive indole, methyl red, and catalase. Meanwhile all isolates were negative oxidase, urea hydrolysis, citrate utilization, Voges-Proskauer and didn’t produce H_2_S.

The incidence of *E. coli* isolation in chicken was 34%. The serotyping of isolated *E. coli* recovered from different organs of chickens revealed that 24 strains could be identified serologically. They belonged to 12 different serogroups. The most commonly detected *E. coli* serogroups isolated were O128 (4 isolates), O111 (3 isolates), O44 (3 isolates), O158 (2 isolates), O2 (2 isolates), O115 (2 isolates), O20 (2 isolates), O29, O15, O169, O125, O26 and O6, while 10 strains were not typed due to antiserum availability.

### Antibiogram pattern of isolated *E. coli*

Antibiogram pattern of *E. coli* in our study revealed that 100% of the isolates were resistant to ampicillin, erythromycin, and sulfamethoxazole/trimethoprim, while *E. coli* isolates were sensitive for colistin sulfate and levofloxacin with 73.53% and 38.23% as shown in Table-3.

**Table-3:** Antibiotic resistance pattern of isolated *E. coli*.

Antimicrobial agents	Resistance	Intermediate	Sensitive
		
	n (%)	n (%)	n (%)
Ampicillin	34 (100)	-	-
Amoxicillin	33 (97.06)	-	1 (2.94)
Gentamicin	22 (64.71)	2 (5.88)	10 (29.41)
Streptomycin	33 (97.06)	-	1 (2.94)
Erythromycin	34 (100)	-	-
Amoxicillin-clavulanic acid	32 (94.12)	-	2 (5.88)
Doxycycline	30 (88.24)	-	4 (11.76)
Tetracycline	32 (94.12)	-	2 (5.88)
Nalidixic acid	30 (88.24)	-	4 (11.76)
Levofloxacin	9 (26.47)	12 (35.29)	13 (38.23)
Colistin sulfate	7 (20.59)	2 (5.88)	25 (73.53)
Trimethoprim-sulfamethoxazole	34 (100)	-	-

### Detection of the genes of isolated *E. coli* by conventional PCR

Screening the presence of *tetA*, *qacED1* and *qacA/B* genes by PCR technique after DNA extraction revealed that *tetA* gene in 31 (91.18%), *qacED1* gene in 24 (70.56%) and *qacA/B* gene in 5 (14.7%) out of 34 tested *E.coli* isolates.

## Discussion

Avian colibacillosis is an extraintestinal infection that can progress into several lesions in diverse organs as polyserositis, cellulitis, salpingitis, perihepatitis, peritonitis, septicemia, airsaculitis, and death. These cause harsh economic losses in the poultry industry, due to the significant number of morbidities, mortalities, slaughter condemnation, and reduced productivity of affected birds [16].

The incidence of *E. coli* isolation in chicken was 34% These results are agreed to some extent with that obtained by Ashraf *et al*. [17] who isolated *E. coli* in Egypt at 38%.

The most commonly detected *E. coli* serogroups were O128, O111, O44, O158, and O2. *E. coli* serotypes had been previously isolated from chicken and newly hatched chicks in Egypt as reported by Ashraf *et al*. [17] who detected O78 and O111, El-Haleem [18] and Taha [19] detected O2, El-Jakee *et al*. [20] collected *E. coli* serogroups O2, O6, O8, O26, O27, O78, O86, O111, O128, O157, and O136 from chicken cloacal swabs, El-Sayed *et al*. [21] founded O111, O55, O142, and O128, El Jakee *et al*. [22] collected *E. coli* isolates serogroups O125:K70, O1:K-, O146:K-, O26:K-, O78:K80, O126:K58, and O128:K67 from diseased chickens to prepare a potent *E. coli* vaccine to control colibacillosis in chickens, and also Bakheet *et al*. [8] identified O2:H6 (2 isolates), O163:H2 (2 isolates), O128:H2 (3 isolates), O158 (2 isolates), and O44:H18 (2 isolates).

Antimicrobial resistance has become a worldwide problem, and the vast consumption of antibiotics by both humans and animals leads to the development and spread of a large number of antibiotic resistance among bacterial populations consequently creating critical public health problems. In the current study, isolated *E. coli* revealed that 100% of the isolates were resistant to ampicillin, erythromycin, and sulfamethoxazole/trimethoprim (each) as shown in Table-3 that results agreed with Subedi *et al*. [23] who showed that the maximum resistance of 50 *E. coli* strains to ampicillin (98%), Bakheet *et al*. [8] who recorded resistant to sulfamethoxazole/trimethoprim 100%, and Radwan *et al*. [24] who discussed that antibiogram profiles of *E. coli* isolates and indicated maximum resistance to ampicillin (100%); furthermore, Eid *et al*. [25] reported that the highest resistance rates were recorded against trimethoprim sulfate, doxycycline, tetracycline, and amoxicillin (94.1%, 93.2%, 92.9%, and 92.3%, respectively). While *E. coli* isolates were sensitive for colistin sulfate and levofloxacin with the percentage of 73.53% and 38.23%, respectively, Makhol *et al*. [26] found that 69.4% of *E. coli* isolates were sensitive to colistin sulfate.

The extensive and prolonged use of tetracycline in the poultry industry is undoubtedly one of the explanations for the high prevalence of resistance to tetracycline in broilers [27]. Concerning tetracycline resistance, in our study, *E. coli* isolates were 94.12% resistance to tetracycline antibiotics.

The *tetA* gene was tested for isolated *E. coli* to assess its resistance to tetracycline. Interestingly, the positive PCR percentage (91.18%) was high as shown in isolates. However, the phenotypic antibiotic susceptibility test was 94.12%, which may be related to more genes than *tetA* gene contributing for tetracycline resistance in *E. coli*. Sengeløv *et al*. [28] examined *E. coli* isolates from diseased and healthy broilers for the presence of tetracycline resistance genes *tet* *(A), (B), (C), (D)*, or *(E)* and found that the *tetA* and *tetB* were the most prevalent; in isolates from healthy broilers, *tetA* was present in 41.2%, *tetB* in 52.9%, and *tetD* in 5.9%, and in isolates originated from diseased broilers, *tetA* was present in 72.2% and *tetB* in 27.8% samples. Furthermore, Abo-Amer *et al*. [29] recorded that tetracyclines genes *tetA* and *tetB* were observed at the prevalence of 65% among *E. coli* isolated from chicken farms in Saudi. There was a correlation between the presence of integrons and resistance to tetracycline in chicken *E. coli* isolates from the Veterinary Antimicrobial Resistance Surveillance Network [30].

QACs are cationic surface active detergents extensively used in the poultry industry as of their low relative toxicity, good antibacterial properties non-irritating, non-corrosive, low toxicity, and reasonably effective in the presence of organic matter. Hence, it makes a disinfectant of choice for equipment such as incubators and hatching trays [31]. Genes that confer resistance to QACs are *qacE* and *qacED1*; *qacED1* a mutant version of *qacE* appears to be partially functional as a multidrug transporter and is widely distributed throughout Gram-negative bacteria due to its location on the 3´ conserved region of class 1 integrons [32].

In this study, the *qacED1* gene was reported in 70.6% *E. coli* (24 positive samples from 43 *E. coli* isolates). These results were nearly in accordance with Amira [33] and El Tawab *et al*. [34] who found *qacED1* gene among *E. coli* isolates (93.1% and 63.16%, respectively) in Egypt. *QacE* gene (including its attenuated variant *qacED1*) is widely spread in Gram-negative bacteria, mainly in *Enterobacteriaceae* [35,36].

*QacA/B* gene was founded in 14.7% *E. coli* in our study; nevertheless, *qacA/B* was founded in Gram-negative bacteria like *E. coli*. It seems that the presence of the *qac* genes does not necessarily imply increased resistance to antiseptics that could be relevant for practice [37].

Antimicrobial resistance has become a worldwide problem, and the massive usage of antibiotics by both humans and animals leads to the development and spread of a large number of antibiotic resistance among bacterial populations consequently creating critical public health problems. The co-resistance of QAC and antibiotics could be attained by linkage of different resistance mechanisms on the similar plasmid, transposon otherwise integrin, or any combination of these [4]. The localization of these QAC determinants on different mobile elements may share in the transmission of resistance to the other bacteria [38]. Among Gram-negative bacteria, the *qac* genes are often related with plasmid-mediated class 1 integrons which harbor a diversity of antibiotic resistance genes [7].

## Conclusion

*E. coli* is one of the most dangerous pathogens that threaten the poultry industry in Egypt due to the high rate of its presence in the farms as well as the presence of the *qac* resistance gene and antibiotic resistance gene in *E. coli* definite a link between antibiotic and disinfectant in possible that needs further study.

## Authors’ Contributions

The study was designed by SAM, SAN, and JKE. WAI and AME did the molecular work. Data collection, analysis and manuscript preparation by WAI. All authors read and approved the final manuscript.
